# Selections and Abstracts

**Published:** 1905-06

**Authors:** 


### SELECTIONS AND ABSTRACTS.

### Briefs on Genitourinary Surgery. Gonorrheal Rheumatism.

By G. Frank Lydston, M.D., Chicago, Ill.

Professor of Genitourinary Surgery and Syphilology State University of Illinois; Attending Surgeon St. Mary's and Samaritan Hospitals.

Gonorrheal rheumatism is a relatively infrequent complication or sequel of gonorrhea. Its existence is disputed by some surgeons, but the majority of authorities admit that certain individuals, who are oftentimes apparently free from predisposition to ordinary rheumatism experience pain and tenderness of the joints, attended with more or less constitutional disturbance and synovial effusion, in the course of gonorrhea, while in others the process attacks various tendinous and ligamentous structures. Some patients are affected with this complication with every attack of specific urethritis. It rarely begins during the acute stage, being most likely to occur after the disease has become chronic. The writer has known it to occur, however, within three days after the onset of the disease.

Few diseases have been the source of more controversy regarding their origin than has gonorrheal rheumatism, but as yet its pathology must be regarded as unsettled. It does not seem to depend upon atmospheric conditions, overexertion, or, it is asserted, on the method of treatment of gonorrhea, the latter proposition, however, being open to doubt. The affection sometimes follows abortive treatment by silver solutions with suspicious rapidity. The relation of the disease to inflammation of the urethra is all that has so far been proved. Actual suppuration of the mucous membrane is by no means necessary in its causation. It has been alleged that, inasmuch as it occurs independently of the ordinary causes of rheumatism, and occurs in such a small proportion of cases of urethritis, it is due to idiosyncrasy. There may be some truth in this view.

The most universal opinion is that gonorrheal rheumatism is a mild sort of systemic infection by the products of urethral inflammation. According to some, the disease is due to secondary infection, the gonococcus having been found in arthritic and tendinous effusions. It is probable that toxins elaborated by the virulent germ infection enter the circulation and finally reach the affected parts. The joint tissues are sometimes extraordinarily sensitive, and arthritic effusion occurs. Whether gonococci or the toxins of mixed infection are the exciting cause of the affection is unsettled. Pus microbes probably cause the exceptional cases characterized by suppuration. General pus infection has resulted from gonorrhea; thus Charteris,* in 1876, reported the case of a youth who died of pyemia on the sixth day after developing virulent urethritis. General gonococcic infection with endocardial involvement has been observed, and verified post mortem. Certain elements in the treatment of gonorrhea may be the primary cause. The destructive effect exerted upon the epithelium of the urethra by strong injections and the rude introduction of instruments has already been noted. If the mucous membrane be abraded, absorption of organic poisons is facilitated. Absorption does not readily occur through the intact mucous membrane, when inflamed. The facility of absorption in certain individuals may explain their special susceptibility. The immunity of women is suggestive. The only explanation of this immunity is the relative toughness of the vagina and endometrium. When the disease does occur in the female, the urethra and bladder will be found to be involved. Rheumatism does not usually follow primarily simple urethritis, showing that the degree of virulence is an etiologic factor. Whether gonorrheal rheumatism is always due to gonococcic urethritis is open to question. The disease arises in cases where there is no evidence of specificity, in spite of the severity of the urethral disease.

I believe that cachectic, strumous, gouty and rheumatic patients are more predisposed to the disease than healthy persons.

*Symptoms:* The disease resembles rheumatic gout rather than ordinary rheumatism. The local evidences of the disease are not usually severe, or the constitutional symptoms marked; but this is

*British Medical Journal.

not always true. The disease comes on, as a rule, during the decreasing stage; sometimes during the second or third month. Some authorities assert that it usually develops in from five to sixteen days. Most cases observed by me have occurred later, although, as above stated, one case developed within three days after the onset of urethritis. In another case only a week had elapsed. The explanation that suggests itself is that inflammatory thickening inhibits absorption to a certain extent. Then, too, epithelial abrasion is not so apt to occur within the first few days as later on, when the infectious material has been in contact with it for some time. The products of the purulent process are not so virulent in the first few days as afterward.

There is usually no change in the urethral discharge coincidentally with the development of the rheumatism; it may be lessened, probably because the arthritis compels the patient to keep quiet. In some cases cessation or marked diminution of discharge occurs and can not be explained. This is not likely to benefit the urethritis. It is doubtful whether gonorrheal rheumatism ever acts as a revulsive or derivative upon the urethral inflammation. It is said that when the rheumatism comes on late there is liable to be an increased discharge for a few days. The *propter* and *post* are probably confounded. It is more probable that from some particular cause exacerbation of the urethritis occurs, with a coincident rapid formation of its characteristic toxic products; the mucous membrane of the urethra being extensively abraded at this time, absorption readily occurs.

The seat of gonorrheal rheumatism varies in different individuals, sometimes in the same patient in different attacks.

The structures involved, in order of frequency, are: (1) The articulations; (2) the synovial sheaths of tendons and muscles; (3) synovial bursæ and the sheaths of nerves. Associated with the latter form inflammation of the pericardium, endocardium, the cerebral meninges, and the deeper structures of the eye may occur. I have seen a number of cases in which the eye alone was involved. There is a marked predilection for the more important joints, the knee being most often affected. As a rule, the inflammation is most severe in one joint, although, in most instances, several joints are eventually involved.

The practical differentiation of cases of gonorrheal rheumatism comprises three varieties, viz.:

1. Generally uniarticular inflammation, most often of the knee, sometimes the ankle or elbow. This is a passive, insidious hydrarthrosis with much effusion. Pain, tenderness, redness, heat, and constitutional disturbance are absent or moderate. Resolution is very gradual, and usually takes some months. Ankylosis may occur. The uniarticular form is sometimes excessively painful and attended by marked constitutional disturbance. It tends to affect the bones of the articulation secondarily. The fluid in such cases is likely to contain more or less purulent material.

2. A variety resembling articular rheumatism. This is accompanied by moderate local and constitutional symptoms. Several points are usually involved; the tendons, various other fibrous structures, and the eye are often implicated. The symptoms are milder than those of acute rheumatism, very mild considering the apparent magnitude of the processes.

The disproportion between the general and local symptoms is important in differential diagnosis. The involvement of the joints is usually consecutive, but there is no articular delitescence, as in acute rheumatism. The profuse sweating, acid urine, and excessive plasticity of the blood, characteristic of inflammatory rheumatism, are usually absent in the gonorrheal variety. The serous membranes, the pleura, endocardium and pericardium are not often attacked. A favorable result is usual, but chronic synovitis, tuberculous arthritis in certain subjects, joint stiffness and complete ankylosis are possible sequels. Fournier affirms that acute suppuration does not occur; it is, however, occasionally seen. A joint may contain considerable purulent fluid and still recover without radical surgical interference. This is not likely in ordinary suppurative arthritis.

3. (a) Indeterminate transitory pains in various joints without either local or general evidences of disease. There is apt to be exacerbation of pain and perhaps distinct involvement of joints coincidently with increase in the urethral inflammation.

(b) Thecitis, the synovial sheaths becoming swollen and tender, perhaps with moderate redness along the affected tendon. Movement of the corresponding muscle is very painful. Synovial bursæ may become involved; those under the tendo Achillis and the inferior tuberosity of the os calcis are most susceptible. Patients thus affected complain of pain and tenderness in the heel. This symptom is not rare in gonorrhea.

Gonococci have been found in numerous forms of gonorrheal rheumatism. Myalgia and perineuritis sometimes occur in gonorrhea. Pain in the back of a severe character, simulating acute lumbago, is very frequently seen. This is usually due to overstimulation of the kidneys by various balsamic preparations, especially sandalwood. It occurs, however, where such drugs have not been taken. Whether reflex nephralgia explains these latter cases is an open question.

Cases are met with where gonorrheal rheumatism limits itself to a single nerve. One of my patients has sciatica with every attack of gonorrhea. Even simple urethritis brings it on in this patient. The first attack of gonorrhea that he ever experienced was attended by severe sciatica, involving both nerves.

It is remarkable that authorities so universally concede the comparative painlessness of gonorrheal rheumatism when many cases are attended by severe pain and not infrequently by sweats quite as profuse as those of ordinary acute rheumatism. The tendency to sweating seemed to be most marked at night, being characterized by absence of acidity, its prostrating character, and great profuseness.

*Treatment:* The treatment of this complication is not satisfactory, being usually palliative rather than curative. The treatment for urethritis should usually be continued; the sooner the local condition improves, the sooner the rheumatism will yield. If, however, the discharge has ceased, it is best to let the urethra severely alone. Urinary antiseptics are always indicated. Debilitated patients require tonics, such as strychnine, arsenic, iron, and cod liver oil. The skin and bowels should be kept active and elimination favored by the hypodermic use of pilocarpine. Pain may demand opiates, hot applications, and fixation of the inflamed joint. Should the knee be severely involved, Buck's extension apparatus should be applied. The application of fifteen or twenty leeches to the joint will often prove serviceable. Flannels wrung out of hot water and sprinkled with turpentine are useful. As the inflammation subsides, blisters or iodine will be found to promote resolution. Mercury and potassium iodide internally are of great service in the chronic stages. I have had excellent results from intraarticular injections of iodoform emulsion. It is well to try the salicylates; the rheumatic or gouty diathesis may be a predisposing cause. The oil of wintergreen often seems especially effective. The larger joints, such as the knee, demand the plaster-of-paris bandage so soon as the acute inflammation has subsided. Passive movement, and perhaps measures to break up ankylosis, are required later. Turkish baths, static electricity, friction and massage are serviceable adjuvants. Static electricity is particularly beneficial. This remedy is of great value in obstinate cases of chronic enlargement of joints. During the acute stage of gonorrheal rheumatism, a milk diet is very essential. Arthrotomy, with or without erasion of the joint surfaces, has proved effective in a number of cases of severe gonorrhea of the knee joint. It is always to be seriously considered in such cases, and will sometimes preserve joint mobility.—*St. Louis Medical Review.*

---

### Note on Chalybeate Therapy.

By William Krauss, Ph.G., M.D.,
Memphis, Tenn.

There have been volumes written on chalybeate therapy and the controversies upon the absorbability of this or that form of iron have occupied the attention of clinicians for many years. Among the many preparations claiming the attention of clinicians, the one devised by Dr. Gude, chemist, of Leipzig, has received the endorsement of the ablest men on both continents for ten years. The writer has used it to the exclusion of all others, with the exception of a few disappointing experiments with others claiming equal merit, and has published two papers upon the subject.

It would seem that when one has used a certain medicament for years and with uniformly good results, and especially if this agent definitely eliminates the shortcomings of formerly employed products of the same elements, one is apt to take its effects as a matter of course, and further discussion would seem useless, or at least superfluous. When it happens, however, as is usually the case after a certain article has had a successful career, that many similar products strive to take the place of the original, generally resulting in disappointment to the user, it becomes a duty to take stock of the evidence in the case and see how far the confidence in the one and the distrust of the other is justified.

For this reason I have had compiled from separates and reprints some of the results of a few trustworthy observers.

1. Secondary Anemia.—H. D. Peterson (*Chicago Medical Recorder, 1896*) treated many cases, reporting five in detail, and says: "Pepto-Mangan is easily absorbed by the digestive tract without any disturbance of the same, is not injurious to the teeth, and produces no constipation."

Dr. J. W. Frieser, Vienna (*Therapeutische Monatshefte, February, 1902*), reports treating many cases, among which he made detailed studies upon forty-three cases and summarizes: "It can be warmly recommended for extensive use in the treatment of anemic conditions."

Dr. Hugo Summa, St. Louis (*N. Y. Medical Journal, 1895*), recommends its use after tests in thirty-four cases, saying: "It is especially worth mentioning that no bad after-effects could be detected. In this connection I call special attention to the absence of constipation that could be traced back to the use of this preparation."

Dr. Sam'l Wolfe, Philadelphia, reports upon fifty cases observed during about four months, and concludes: "That Pepto-Mangan is a highly available preparation of iron, on account of its liquid form, pleasant to taste, non-corrosive action on the teeth and unirritating effect on the digestive organs, admitting thus of easy gradation of dose, easy administration to children and avoidance of unpleasant effects in all cases. That it is an efficient and rapid restorer of the normal quantity and quality of the blood, etc."

Dr. Fritz Euler-Rolle, Vienna (*Wiener Klin. Rundschau, March 29, 1903*), mentions fourteen cases of anemia, besides a number of cases of other diseases, in a very complete report in which he is pleased with its absorbability and (on account of the abundance of peptone it contains) its food value in delicate stomachs, and finds it free from all the objections usually urged against iron preparations, and its results prompt.

Dr. C. A. von Randohr (*N. Y. Medical Journal, June 26, 1897*) in connection with some gynecological cases, reports seven cases of anemia in which there was a rapid improvement.

Dr. H. P. Loomis, in a paper before the New York Academy of Medicine (June 18, 1893), reports a number of cases, eight in detail, in whom there was a rapid increase in red cells and hemoglobin, and in most cases with no constipating effect.

Drs. Diago and Benitez, Superintendent and Chief of Laboratory, Hospital No. 1, Havana, Cuba (*Progreso Medico, Havana, April, 1902*), report six cases in detail and summarize as follows: "We may say conscientiously that it is the best remedy we know of for the purpose, and that we do not hesitate to commend it to the profession, especially our confreres in Cuba, as an iron preparation that possesses all the advantages that can be demanded of such a remedy and none of the disadvantages that are characteristic of other iron preparations. We would especially emphasize also that Pepto-Mangan (Gude) is very pleasant to the taste, and is most easily taken by patients of all ages and with the most delicate digestions."

Dr. Juan Pablo Garcia, Havana (*La Revista Medica Cubana August 1, 1902*), says: "I have had the opportunity of testing the efficiency of this preparation of iron in a large number of cases in both hospital and private practice, and have found it the most satisfactory iron compound that has come under my notice. It is not at all constipating, its taste is not astringent, so that it lacks the great disadvantages of most other iron compounds." He says further that it causes no disturbances of digestion and its therapeutic efficiency has been attested by the best clinicians throughout the world.

Dr. Louis J. Gravel, Physician-in-Chief to the Hotel Dieu, Montreal, (*Buffalo Med. Jour. August, 1903*), prefers the term dysemia, meaning "bad blood," reports thirteen cases with blood examination, and says : "Comparing my results with Pepto-Mangan (Gude) with those obtained from other chalybeates of this class, I have been led to give it decided preference."

Drs. Chibas and George A. de Santos Saxe, of Columbus Hospital, New York (*Int. Jour. Surg., June, 1903*), report forty cases, with tabulated results and complete bibliographic review (which has facilitated this compilation very much), and say they have used it in the hospital for over two years in anemic convalescents with uniform satisfactory results. "In no case did constipation, nausea, headache, or digestive difficulties follow its administration."

Dr. Hermann Metall, Assistant Physician at the General Polyclinic, Vienna (*Med. Chir. Centralblatt, January, 1902*), reports twenty-three cases of anemia consequent upon a variety of condition, of which twelve showed a normal hemoglobin per cent. after fourteen days, five after three weeks and five after a month. He concludes that it is "a reliable and valuable blood-building remedy, which can be recommended for general use in appropriate cases."

Anemia Consequent upon Special Conditions.—Dr. Mateo M. Guillén, at Randall's Island Children's Hospital, gives a tabulated report of thirty-two cases of infantile anemia with very elaborate blood-chart, including some very desperate cases of cachexias, and says: "In no case did we have to suspend treatment on account of any untoward influence on the delicate organism of sick infants." Three cases classed as hopeless made a complete recovery. The paper is very instructive.

Dr. J. M. Frieser (*vide supra*) also reports great success in infantile anemia.

Dr. J. K. Bauduy (*St. Louis Medical Review, February 26, 1898*), reports upon a number of cases of neurasthenia, twelve of them in detail, with blood examinations by Dr. Carl Fisch, and says: " . . . the results, however, were indeed a surprise to myself, for the concomitant deranging sequelæ were so slight that but in very few instances in my extensive utilization . . . was I obliged to discontinue it. . . . this particular remedy, I am now convinced, will prove a great boon to the patient and the physician. . . . of course we do not consider the remedy applicable to cases of lithemic neurasthenia, nor in any manner a specific in any variety of neurasthenia."

Dr. Ludwig Pohl, City Physician, Vienna (*Aertzl. Centr. Anzeiger, September 20, 1899*), has used Pepto-Mangan (Gude) in over one hundred cases of chlorosis, anemia, neurasthenia, hysteria and malarial cachexia, and says: "I have previously mentioned that it may be positively assumed that Pepto-Mangan (Gude) stimulates the hemopoietic organs to increased activity. . . . . Decided amelioration in the leukemic state, arrest of the process in severe cases for a long time, reduction of the glandular swellings, improvement in the relation between the red and white corpuscles, were noted by me in several cases."

Dr. J. S. Perekhan (*Chicago Clinical Recorder, 1896*), reports a number of cases, six in detail; Dr. C. A. von Ramdohr (*vide ref. supra*) twelve cases in detail; and Dr. Gellhorn, at Mackenroth's Clinic, Berlin (*Therap. Monatshefte, 1897*), mentions sixty cases, some in detail, all of gynecological patients with a variety of conditions, some post-operative, and all testify to the improved blood findings, Dr. Gellhorn concluding as follows: "I feel justified in asserting that in my therapeutic trials with Pepto-Mangan I obtained all that can be rationally demanded."

In surgical conditions calling for improvement in the blood, Drs. Stuart McGuire (*Vir. Med. Semi-Monthly*), twenty cases, and George G. Van Schaick (*N. Y. Med. Journal, June 2, 1900*), fifty cases, report favorable result. The latter concludes: "We have in such a preparation as Pepto-Mangan (Gude) a means of obtaining good results with a certainty that is almost mathematical, and without any of the distressing symptoms so frequently following the use of the inorganic iron preparations."

Dr. H. Edwin Lewis, Burlington, Vt. (*Vt. Med. Monthly*), Dr. Ed. C. Hill, Denver, Dr. W. O. Davis (*N. Y. Int. Journal, Surg., September, 1902*), together with some of the authors already mentioned call especial attention to the value of the preparation in irregular menstruation, sterility and other sexual anemias in women.

Dr. Karl von Ruck (*N. Y. Medical Journal*) finds in Pepto-Mangan (Gude) the best preparation in the anemia of tuberculosis, being more efficient and more easily borne; he had used it in seventy cases, twelve being reported in detail, in some of which comparative tests would be made with other iron preparations. Other observers also mention it in tubercular anemia.

Cachexia finds especial mention by Fritz Euler Rolle, Pohl, and Fasano, the latter professor at the Royal University, Naples (*Arch. Int. di Med. e Chir., March, 1899*), discusses iron medication in detail, calling special attention to the chemistry of the subject and reports having treated primary anemia, twenty cases; chlorosis, twenty-five cases; malarial anemia, seven cases; tubercular anemia, eight cases; uterine diseases, eleven cases; scrofulosis, twelve cases, rachitis, ten cases; convalescents of exhausting diseases, fifteen cases; total, one hundred and eight cases. He says: "To recapitulate, Pepto-Mangan (Gude) not only deserves the place it has already acquired in therapeutics, but it merits even greater recognition, because all clinicians ought to make use of it in pathological processes in which the object is to restore to its normal condition the altered quality of the blood."

After this array of evidence it is only necessary to add that since this constitutes about all the clinical literature upon which our knowledge of the subject is based, it is rather significant that it was all done upon this particular product. In no instance is it claimed that the preparation is as good as something else, but it is simply set forth by the observers that this was the preparation used and that it met these indications in the manner described. Since there is no official preparation that meets these requirements the manufacturers of Pepto-Mangan (Gude) deserve all the credit which the product has earned for them.—*Charlotte Medical Journal, February, 1905.*

---

The first of the German sanatoriums founded by Prince Hohenlohe in the island of Madeira has lately been opened. It is situated at a height of 300 metres above the sea level, and has accommodation for sixty patients.

### THE TESTS FOR OCCULT BLOOD IN THE FECES AND THEIR CLINICAL SIGNIFICANCE.*

By J. DUTTON STEELE, M.D., Philadelphia.

[Preliminary Note from the Clinical Laboratory of the Presbyterian Hospital.]

In 1901 Boas first announced the possibility of recognizing minute quantities of blood in the stomach contents and feces by means of a modification of the old guiac-turpentine reaction. However, until last winter the method did not excite the interest that the subject appears to deserve.

In the fall of 1903 Boas published another paper dealing with his results with the tests in a series of cases of gastric ulcer, and this was closely followed by a paper by Hartmann which considered the whole subject in a most comprehensive manner. The tests are practical and very easily applied without special apparatus.

If the reaction is everything that is claimed for it, and it has already stood successfully the tests of many series of clinical observation, it is one of the noted advances in medical science of recent years and an extremely important addition to our methods of diagnosis in diseases of the gastro-intestinal tract. In a limited experience with the practical working of the reaction I am convinced that it will take a place of importance in the diagnosis of gastric ulcer and cancer comparable with the absence of hydrochloric acid in cancer and with hyperacidity in ulcer. I have therefore taken the opportunity of presenting the matter to the society with a short review of the test and of a preliminary note of my experience with it.

As originally used the test was intended to detect blood in the stomach contents, but it was found that the unavoidable abrasions caused by the stomach tube in removing test meals caused enough bleeding to give the reaction even in normal stomachs. At the same time it was discovered that the test was applicable to the feces quite as readily as to the stomach contents, and at present attempts to discover occult blood are entirely confined to the feces.

*Read at the meeting of the Medical Society of the State of Pennsylvania, held in Pittsburg September 27-29, 1904.

The clinical significance of occult blood in the feces is practically the same as the presence of large hemorrhages. But the great delicacy of the test enables us to detect bleeding from ulceration in the gastro-intestinal tract, malignant or otherwise, much earlier in the course of the disease and before the occurrence of massive hemorrhage has sapped the patient's strength and complicated the case.

Moreover the test offers an excellent indication of the course of such diseases. Thus it can be a control for our cases of gastric ulcer, which are undergoing the so-called ulcer cure. For instance, Boas keeps his patients at rest on a milk diet until occult blood has disappeared from the stool, indicating the healing of the ulcer.

Briefly the test is as follows:

1. Hemorrhoids, menstruation and kindred sources of hemorrhages must be excluded.

2. It is probably better to exclude hemoglobin in the form of meat or meat juices from the diet for a day or so before the test is applied, though authorities differ as to whether the remains of cooked meat in feces will or will not give the test. I have not definitely settled this point to my own satisfaction, but my observations, up to the present time, indicate that moderately well-cooked meat will not give the reaction in the stool. The stool should be made soft by some mild saline.

3. A small portion, about five c. c. of soft or softened stool, is abstracted with twenty c. c. of ether to remove the fat and fatty acids which interfere with the test.

The ether is allowed to rise and is poured off. A small beaker is very convenient for the test.

Two or three c. c. of glacial acetic acid are added to the feces and the whole is stirred thoroughly. Then this mixture is again abstracted with ten c. c. of ether. This ethereal extract is the portion to be tested. About two c. c. of this is taken in a test tube and two or three drops of a freshly prepared tincture of guiac are added. Instead of the tincture a little of the resin can be added directly to the ethereal extract in which it dissolves readily. Then add twenty to thirty drops of pure hydrogen peroxid, and shake.

If blood is present the ethereal extract will turn a clear blue which may appear a purplish blue if the ethereal extract is brownish, as it often is. The color fades quite rapidly. Ozonized oil of turpentine may be used instead of the hydrogen peroxid. It is absolutely essential that the turpentine should be chemically pure and should be ozonized by allowing it to stand exposed to the air for four to six weeks. In my laboratory work I have used Mercks' oil of turpentine, which has proved very satisfactory for the purpose. In my experience, hydrogen peroxid gives a prompter and more delicate test with the guiac than does the ozonized oil of turpentine, although the latter acts much better than the aloin test.

The second test of the detection of blood in the stool is the aloin test of Klunge. It is made as follows:

A fresh solution of aloin is prepared by dissolving as much aloin as can be taken on the tip of a spatula, in ten c. c. of seventy per cent. alcohol. The ethereal-acetic extract of the feces is prepared in the same way as in the other tests. To two c. c. of the ethereal extract an equal quantity of the aloin solution is added and then ten to fifteen drops of either ozonized oil of turpentine or hydrogen peroxid is dropped in drop by drop. Here the turpentine acts much better and more delicately than does the hydrogen peroxid. If blood is present the lower half of the fluid in the test tube will turn a rich cherry red after standing for a short time, while in the absence of blood it will remain a light yellow. I have used this test with great satisfaction as a control for the guiac reaction, and in my hands it has seemed quite as delicate.

In my laboratory experience I have been accustomed to make all four tests, that is, guiac-turpentine, guiac-hydrogen peroxid, aloin-turpentine and aloin-hydrogen peroxid.

Since the summer I have made 265 separate tests in forty-eight cases. My results have conformed very closely to those given by the German observers. They can be summarized as follows:

1. In cancer of the stomach or intestines the test has shown blood in almost every stool passed.

2. In ulcer of the stomach, blood is not as a rule found in every stool, but is irregularly present. Sometimes it may miss one or two or even more stools.

Under this division of intermittent occult bleeding comes also duodenal ulcer, benign organic pyloric stenosis and spastic pyloric stenosis.

3. Occult blood is never found in gastritis acida, anacida or subacida, hyperacidity, hypersecretion (without ulcer), benign dilatation and neurosis.

It will be seen how valuable the test may be, for instance in the distinction between a painful neurosis and a gastric ulcer, or a chronic gastritic anacida and carcinoma. My own experience has so far confirmed what the Germans say concerning its value. In every case the subsequent course of the case confirmed the diagnosis suggested by the finding of occult blood. The following series of cases illustrate the availability of the test:

Case one was one that had been operated upon for pyloric stenosis and in which extensive carcinoma was demonstrated. It gave a positive test.

Case two was a gastritis anacida with suspected carcinoma. Test, negative. Subsequent course was against cancer.

Case three was a woman in the menopause, whose history strongly suggested gastric ulcer—with pain, vomiting and a history of some hematuria. The test was always negative. Later vomit was examined and showed a subacidity. A diagnosis of gastric neurosis was made which has been confirmed by the course of the case.

Case four was a well-marked case of ulcer ventriculi. Tests generally positive.

Case five, enlarged liver with jaundice. Positive test. Diagnosis lies between cholemic bleeding and hypertrophied cirrhosis or cancerous ventriculi with secondary involvement of the liver, or cancer of the bile-ducts.

Cases six and seven, gastritis acida and anacida. Test, negative; no signs of ulcer or malignancy.

Case eight, ulcer presumably healed; negative.

I am so convinced of the clinical value of the test that I am glad to have the opportunity of bringing it to the notice of those members of the society who have not had an opportunity of applying it in practice.

### THE CALCULATION OF THE DATE OF DELIVERY IN PREGNANCY.

BY W. J. CAIE, M.A., M.B., B.CH., BURY ST. EDMUNDS.

Though authorities differ somewhat as to the average duration of pregnancy in woman, the amount of divergence is small, and it is generally agreed that there is no absolutely sure method of calculating the exact date of delivery, the causes of this being mainly the impossibility of determining the date of fruitful coitus, combined with our ignorance of the factors which go to induce the occurrence of labor at term. Added to this is the prevailing inaccuracy of the woman regarding the date of the last menstrual epoch.

The length of gestation is given by Matthews Duncan[1] as 278 days, Schlichting[2] 273.2 days, Ahlfeld[3] 281.6 days, Lowenhardt[4] 279.8 days, Hasler[5] 280 days, Montgomery[6] 276 days, Edgar[7] 280 days, giving an average of 278.3 days.

The simplest calculation is, perhaps, that by Naegeli's method, which consists in counting three months back from the date of the onset of the last menstrual period, adding seven days (in Leap Year six days if February be included), and counting a year forward from the resulting date.

I have kept by Naegeli's method a record of two hundred cases which were under my own observation, only those being recorded in which, on inquiry, the woman herself gave an exact date for the onset of the last menstrual epoch, and the results are as follows :

| | | |
|---|---|---|
| A. | Labor occurred before the estimated date | ......53.70 per cent. |
| B. | " " after " " | ......24.50 " |
| C. | The estimated date was exactly correct | ......16.04 " |

The remaining percentage is accounted for by immature and premature labors.

In A labor occurred on an average 3.4 days before the estimated date.

In B labor occurred on an average 1.8 days after the estimated date.

There are, therefore, two points about this analysis which should be noted :

1. The percentage of labors occurring before the estimated date is far in excess of those occurring after it.

2. In Class A the average number of days is greater than in Class B, or, in other words, those in which the date of delivery is after the estimated date are nearer the latter than those in Class A.

I argue, then, from these facts that, in Naegeli's method, the number of days added on is too great, and that by adding fewer days a still nearer approximation to the date of delivery might be got, and this conclusion comes into line with the average length of of gestation arrived at from the authorities quoted above, which is less than that given by Naegeli. If two days less be added the result corresponds both to the average length of pregnancy which I have quoted, and with the average (practically) in Class A.

Where available—which, unfortunately, is seldom—Lowenhardt's method will be found to be more accurate than Naegeli's, and the two will be found to correspond almost exactly, provided two days fewer are added to the latter. In fifty other cases which I calculated by Lowenhardt's method, the date of delivery was in all within 1.6 days of the estimated date. Lowenhardt's method consists in counting the number of days between the last menstrual period and the one preceding that and multiplying by ten. The date can then be got by tables.

REFERENCES.

[1] *Arch. f. Gynak.*, Bd. iii, 456. [2] Ibid., Bd. xvi, 210. [3] *Beobachtungen uber die Dauer der Schwangerschaft.* [4] *Die Berechnung und die Dauer der Schwangerschaft.* [5] *Arch. f. Gynak.* [6] *Signs of Pregnancy.* Second edition. [7] *Practice of Obstetrics.*

---

The Japanese, says *American Medicine,* are making every effort to prevent the appearance of disease during the coming warm weather. Thousands of soldiers and Chinese are engaged in cleaning Mukden and the vicinity of the battlefields. The Russians left the city in a very unsanitary condition, and this will result probably in much sickness during the summer unless the sanitary measures of the Japanese are successful. Strict orders have been issued regarding the maintenance of purity of the drinking-water, and other preventive measures will be taken.

---

Dr. William S. Thayer has been elected to the chair of clinical medicine in the Johns Hopkins Medical School.

### TRAUMATIC NEUROSIS.*

BY H. C. RUTTER, M.D., COLUMBUS, O.,
Formerly Superintendent of the Ohio Hospital for Epileptics.

Traumatic neurosis is the *bete noir* of all common carriers, and has been described under a number of different names.

From having resulted so often by reason of railway accidents, it was known as the railway spine, railroad shock; and having been described fully by Erichson, it took his name sometimes and was known as Erichson railway spine; it is also recognized as nervous shock, physical shock, neurasthenia following shock and accident; and also as accident neurosis, etc.

It is protean in its manifestations and so mixed up with purely psychic symptoms, that it has frequently puzzled the most acute diagnosticians, and bothered courts and juries to an unlimited extent. The records of the courts of Franklin county contain numerous cases of this description, and the trials of these cases have given rise to a greater divergence in medical testimony than all other cases combined. It may be divided into two classes; the first having an apparent somatic basis, and the latter appearing purely psychic as far as a physical examination can reveal a lesion.

Of the first class I shall have but little to say in this paper, but confine myself to what is known as hysterical traumatic neurosis, or an hysterical condition presenting all of the subjective symptoms of traumatic neurosis, without any of the physical signs, or at best, very few.

There can be no doubt but that physical injuries have occurred in all cases, but so far they have eluded observation, and can only be judged by subjective symptoms. Nor can this be considered very remarkable when we reflect that long-continued chronic mania, for instance, mania in which the healthy operations of the mind have been destroyed for years, does not leave, in many cases, the slightest change in brain structure observable to the pathologist.

In order to get a true conception of this disease or condition, it

*Read before the Columbus Academy of Medicine, March 6, 1895.

will be necessary to say a word in reference to hysteria. How shall we define that word? To properly do so would consume much more time than is allotted me in this paper, especially if we dealt with its causes, manifold manifestations and relationship to other diseases. I shall, therefore, be content to define it as a condition of the nervous system in which an innumerable variety of manifestations, both mental and physical, direct and reflex, present themselves in various forms, and often in most contradictory sequences, and frequently without any appreciable pathologic basis, and most frequently without any serious danger to life.

This condition constitutes a disease of the nervous system, whether a pathologic basis is found or not. And like all other diseases, whether somatic, or apparently psychic, is not under control of the will and can not be altered by any voluntary act of the patient. This fact must not be lost sight of, and because a shock or sudden fright or clever ruse is sometimes sufficient to relieve many purely psychic attacks of hysteria affords no reason for saying that the whole disease is assumed and therefore voluntary. Or, that when contradictory answers are sometimes given during an examination, and hypersthetic and anesthetic zones suddenly and unaccountably change, that therefore the patient is a base malingerer and that no disease is present.

The condition follows accidents more or less severe, occasionally so slight as to leave no physical evidence, that is to say no injury which can be demonstrated by physical examination. Because a certain number of persons thus affected have figured in damage suits against railroad and other corporations and business firms, a disposition to regard the condition with suspicion has been engendered; and because no somatic or physical foundation is always discoverable, doubts arise as to the honesty of the patients' statements. The fact that innumerable sufferers from this condition have never brought action against any one, and have sustained great losses and much inconvenience and distress because of the diseases, is too often overlooked, and attention directed entirely to the cases brought into public notice by the damage suits. Oppenheim has said the disease is known all over the world, and throughout many parts of the world where damage suits are unknown, even among people where railroads have not yet made their appearance, and where no motive to deceive could possibly exist. With cases presenting unmistakable evidence of injury, we will not concern ourselves in this essay. But the same symptoms complex are present in cases which present no physical evidence of injury, as in those in which the injury is clearly apparent. We are often consulted by patients who have been present in railway or other accidents, and in whom a physical examination fails to reveal any injury; in fact where all the symptoms appear to denote a purely psychic disturbance. In such cases we are not warranted in assuming that the patient has sustained no injury, or that the symptoms are merely assumed or imagined. I have seen many cases in which the losses resulting from inability to work, and the suffering caused by various pains, together with great nervous irritability far exceeded the same conditions arising from fractured limbs or other apparent injuries. And yet if a limb be broken, or a joint dislocated, the corporation responsible never thinks of contesting a claim for reasonable damages, while they persistently refuse to recognize a far more serious condition because it is not susceptible of absolute demonstration as that afforded by the X-ray, for instance.

And this view is too often encouraged by physicians who testify that the condition is nothing but "hysteria," implying thereby that it is self-imposed, or at least under the control of the will. Nothing can be more unjust to a claimant, or more erroneous as a statement of fact.

Traumatic neurosis has been abundantly recognized by the courts, but when coupled with the word "hysteria," is more likely to be regarded as a condition which the patient can cure by an application of the will power alone, and therefore a condition for which the patient is in a large degree responsible. Indeed the term, as usually applied, casts obloquy upon the patient, and is sneeringly and sometimes contumaciously used. A justification of this opinion is sought in the pointing out of sudden recoveries, some of which have followed fast upon the heels of a verdict, or upon the withdrawal of benefits if a member of benevolent organizations. While this may be occasionally true, whether the patient is or is not simulating a disease, it is equally true that a very large number do not recover when every incentive favors such a course. These exceptions only prove the rule as the following case in part will demonstrate; a case which I regard as a typical case of traumatic neurosis without distinctive physical signs of disease. The case was referred to me through the kindness of Dr. Hamblin.

Mrs. V., aet. forty; married; occupation, masseuse; was a strong, active woman, full of life and self-assurance; of a cheerful, jolly disposition; never moody or irritable; never complained of pain anywhere; always had perfect health and laughed at trouble. She met with an accident on the street-cars July 19, 1903. The apparent injuries she received were comparatively slight, so far as a physical examination revealed them, and a careful physical examination was made by very competent surgeons.

I saw her two months later. She was then very irritable and "nervous," easily excited and startled, feared everything, and had lost fifteen pounds in weight; complained of pains distributed at different times over the entire body; frequent headache, sometimes frontal, sometimes occipital, sometimes on the right side, sometimes on the left; frequent intercostal pains, pains of a lightning character in both feet and legs, pains referred first to one thigh and then the other or both; general hyperesthesia, both of the skin and deeper tissues; great weakness of the legs, so that she was obliged to use a cane to assist in walking; tremor of hands and feet; reflexes all exaggerated and over-excited; tinnitus; dizziness; insomnia; flashes of heat followed by cold and chills; burning in palms of hands and feet, and on top of head; difficulty in swallowing; globus hysteria; hypersensibility of the skin as shown by flinching and drawing away at the lightest touch which was observed by her. She was timid and afraid to attempt going out unattended; was afraid to get off and on the cars, and easily frightened by the most trivial circumstances; she sustained noticeable loss of memory and was exceedingly absent-minded; was cross and fretful about her home, her disposition having completely changed since the accident. A physical examination revealed but scant evidences of disease. Heart irritability was increased, the pulse being uniformly over one hundred; the visual field as measured by the perimeter showed a concentric contraction which was confined to the smallest circle, exciting the strongest suspicion of an attempt to deceive, but I was fully satisfied that it was due entirely to a depraved psychic estimate of the objects presented for inspection. She certainly had a much wider field of vision, but her perturbed mental state made it impossible for her to tell when the objects came into her visual field. All the reflexes were exaggerated. There were no anesthetic areas; no paresthesias, excepting only the apparent hyperesthesia everywhere present. The psychic conditions present rendered the use of the dynamometer and aesthesiomometer useless. She could not, apparently, move the dynamometer index a single point, and yet her grip when not noticed was fairly good. The electrical reactions were all normal, and aside from the tenderness over cervical portion of spinal column, together with the rapid pulse, there was no evidence of injury apparent.

Acting largely upon my advice this woman settled with the street railway company for a very small amount, and, according to all known rules of procedure from the defendant's point of view, should have shown a rapid recovery. But although more than a year has passed since the settlement, and she can not hope to get any further assistance from the company, she persistently refuses to get well, and continues to drag out a most miserable existence, her condition growing more distressing each day.

Pain is the most constant symptom of traumatic neurosis, and is not uniform as to character, position or duration. It may be felt in any part of the body, and is of infinite variety. Headaches are usually present and may affect any part of the head or all parts. Sometimes the headaches are associated with vertigo. Hyperesthesia is commonly present and is usually limited to small areas; is mostly incomplete and affects the skin and deeper tissues. The plantar hyperesthesias are very significant of hysterical neurosis. These hyperesthetic areas are often identical with the hysterogenic zones, though not always so.

The zones are found in all parts of the body, but are particularly prominent in the ovarian regions. The ovarian zones are somewhat higher than the ovary, and are found in the same position in men as in women.

The backache is usually combined with hyperesthesia of the skin over the spinous processes, and is more severe than that associated with spinal disease. Its psychic character is easily recognized by the fact that when the attention of the patient is distracted, it does not appear.

The special senses are also hypersensitive. This is shown by the increased ability to recognize faces, and by idiosyncrasies of taste and smell. Oppenheim says the importance of anesthesia as a diagnostic symptom of hysterical neurosis is very great. It is characterized, first, by its manner of extension; second, by its close connection with disturbances of the organs of sense, and in its psychic nature and its reaction to external influences. It does not as a rule follow the distribution of any particular nerves, but appears at random in any part of the body. Sometimes it affects the hand, or arm and hand, or feet or leg; like a glove or stocking, being limited to certain parts of the body regardless of nerve distribution. Hemianesthesia is a favorite form. It is not infrequently limited to the head, the line of demarkation being abrupt, but without regard to nerve distribution. Not seldom also are the special senses, or one of them at least, simultaneously affected, when no connection between the two can be explained by the nerve tracts.

Concentric contraction of the visual field is of importance, not only as furnishing a valuable indication of the character of the disease, but also by assisting in eliminating impost, since it would seem impossible to simulate it, and therefore when found present, as proved by the perimeter, it affords almost unmistakable evidence of the sincerity of the patient's claim for disability. Various forms of motor disturbance have been noted, as well as trophic changes, both in musculature, the skin and other tissues. Paralysis, sometimes local, sometimes general, occurring either in the form of hemiplegia or paraplegia, is frequently present; sometimes absolute, sometimes partial. Pseudo-muscular contractures which yield readily under chloroform narcosis are not infrequent, as well as vasomotor disturbances of the skin which sometimes assume the form of urticaria. In many cases the hair turns gray prematurely, while in other cases there is marked alopecia, the baldness coming on with astonishing rapidity. It must be noted that the word psychic, used so frequently in this paper, is not intended to refer to any intellectual disorder, but rather to a condition, or defect in the character and in emotional disturbances closely correlated to physical symptoms.

We find, also, excessive irritability, manifestations of irritation of mind out of all proportion to the apparent provocation, or without provocation of any kind whatever, rapid variation of emotional states, loss of will power over emotional states, and also certain acts. The mental power is not weakened, but on the contrary is frequently sharpened. There is more or less exaggerated tendency to fear. The patient, not naturally timid, becomes the victim of undefined dread, not only of imaginary dangers, but of the every-day, ordinary dangers of life. For example, if driving they become afraid of the horse; afraid of all sorts of accidents which never caused them a moment's thought before; they dread run-aways, etc., and are miserable during all the time the drive lasts.

The disease has considerable medico-legal importance, as the physician has frequently to assist in the administration of justice, aad must be on his guard to prevent the extortion of money by imposters, as well as to safeguard the interests of those who actually suffer from the effects of this most distressing malady. While the symptoms complex renders the disease easy of recognition by the physician, it is often a most difficult matter to get an opinion before a jury, in contested cases. The declarations of the patients are inadmissable, on the ground that they are "self-serving," and although it would be well-nigh impossible for a patient to group together a set of symptoms so as to form a picture of a recondite disease such as the one under consideration, the medical witness is held to the objective symptoms only, and in many cases these are either wanting or so elusive as not to be capable of development by any instrument of precision we know of at present. It belongs to a group of nervous diseases which present subjective symptoms only, such as can not be measured by any known instruments. If, however, we find a rapid pulse, a contracted visual field, exaggerated reflexes, joined to the symptoms herein detailed, the diagnosis may be regarded as certain and the only question will be as to the permanency of the trouble.

As a rule, the longer the symptoms have lasted the more likely the trouble is to be permanent, although even chronic cases have been known to make good recoveries under favorable circumstances. In cases where nutritional changes have occurred, the probabilities for recovery are not so good. The same may be said in regard to motor paralysis.—*Columbus Medical Journal.*

---

### IRRATIONAL SURGERY, OR SURGERY GONE MAD.

BY BYRON ROBINSON, M.D., CHICAGO.

We live in the era of progress. The signs of progress are the changing of questions and shifting of grounds. Old questions are put into new forms. Old grounds are defended on new principles. The progressive tendency pervades every department of life. Deep in every human heart there lies the hope of progress. Progress consists in lessening suffering and consequently increasing the sum of happiness. It consists in converting Nature's forces into use for man's welfare. There was progress when the light of kerosene superseded that of the tallow candle, when the electric light superseded that of gas, because it saved the eye from strain and facilitated its usefulness. There was progress when the slave was liberated from his bondage, because it allowed him opportunity for development and usefulness. In surgery not every new operation indicates progress, because all are not useful. Some do not lessen suffering or add to the sum of happiness. On the contrary, some new operations have been abandoned because they have proved damaging. Modern surgery appears to have delegated to some unlicensed liberty to invade with impunity any and every organ. To practice their harmful fad operations on the greatest number of victims seems to be their highest ambition. What the final effect of these operations is apparently does not concern them. The past generation has seen the introduction and extensive practice of several notorious fad operations. We shall always have in medicine and surgery the faddist.

### OOPHORECTOMY.

In 1872, Dr. Battey, of Rome, Georgia, introduced the profession to the most useless, disastrous, and destructive of all fad operations. The irrational operation (oophorectomy, or normal ovariotomy) was the removal of the ovaries to "anticipate the menopause," and therefore the nervous woman suffered the irretrievable loss of her chief sexual organ, which is the ovary. For thirty years surgeons have subjected woman to this irrational mutilation of her sexual apparatus with consequent wrecking of her nervous system. Nervous disease was naively mistaken for genital disease by the faddist, and curiously enough this financial mistake of normal ovariotomy has not fully ceased. The ovary is the chief central sexual organ of woman, as the testicle is of man, and should not be removed except for grave conditions or malignancy. It is an essential for women, not only for ovulation, but for internal secretion, to maintain the balance of the nervous system. Fifteen years ago, when I was a pupil of the late Mr. Lawson Tait, one of the greatest surgical geniuses of his age, he was removing healthy ovaries by the score for all kinds of pains and neuroses, with consequent neurotics following in the wake. For twenty-five years the slaughter of the genitals has been rife. Ovariotomy should have been a passing operation from the beginning, but with the ignorant, the knave, and the faddist, it is extensively performed to-day. It is surgery gone mad, for could the operator observe what a wrecked nervous system his surgery had wrought he would cease his irrational procedures. Some are now inventing a medicine so that ovariotomy can be performed and the patient cured of the evil results by taking ovarian extracts or substances.

### TRACHELORRHAPHY.

The next operation, not so disastrous but equally abused, because every tyro thought himself capable of performing it, was Emmet's trachelorrhaphy. Operation on the cervix became a fad almost in every part of the country. Not only general surgeons but general practitioners, essayed to incise the wounded cervix. Almost ten years ago a young practitioner, of perhaps a dozen years' practice, told me he had performed over four hundred cervical operations, almost all of them in his office. Think of this general practitioner performing a trachelorraphy every third day. Is that not surgery gone mad? To-day we know that few surgical operations are required. The contracting, nerve-traumatizing, cicatricial plug in the cervix has lost its terror under rational observation. Dr. Emmet, the inventor of trachelorrhaphy, predicted that the operation would be abused.

DILATOR AND CURETTE.

The days of uterine dilatation and curettage arose, and unfortunately still continue with little abatement. The curette has done more harm than good. Its use is a dangerous, treacherous operation, with limited usefulness. The dilator makes hundreds of atria for infection at every employment. Endometritis that requires the curette is largely a myth; better in general employ an escharotic (carbolic acid) than the curette, as it does not infect.

I have often told general practitioners that if they had a strong iron box with the sound, dilator, and curette, locked in and the key lost, they and their patients would be better off. The dilator and curette may be useful, but in a limited number of cases only. A goodly portion of my income has been derived from the evil results of the general practitioner's use of the dilator and curette, because it has facilitated the transmission of infection through the oviducts into the pelvic peritoneum. The dilator and curette are supreme instigators of pyosalpinx and pelvix peritonitis.

THE PEXIES.

The greatest and most widespread fad of the present day is the pexies—the fixation of normally mobile organs, as in nephropexy, hysteropexy, gastropexy, hepatopexy, oophoropexy. Surgery has certainly gone mad over the fixation of mobile organs. It is a curious madness—the cure of opposites—because the pexies aim to cure a supposed lesion (excessive mobility) by creating another lesion (fixation). Have the pexyites (typical faddists) just discovered that organs are movable? Mobile organs should remain movable; they should not be fixed. It is unphysiological to fix a mobile viscus. Fixation is permanent dislocation. Who fixes joints? The abdomen is a joint and no part should be fixed. The common functions of the chief abdominal organs are peristalsis, absorption, and secretion, and fixation comprises these functions. Fixation comprises the circulation of blood and lymph, traumatizes the nerve periphery, nourishment becomes defective, and structure becomes distorted, while physiology becomes pathologic. James Israel, the world's most distinguished renal surgeon, says that he fixes the kidney or performs nephrorrhapy for two reasons only, viz.: First, to save life; second, for periodic hydroureter. I wonder if the vast number of nephropexies recently performed have been put to Israel's test. Surgeons get daft on fad operations from ignorance, knavery or gain, abusing the science of surgery.

Is nephropexy safe? A short time ago a young Chicago woman died a week after nephropexy was performed. As a gynecologist we assert that hysteropexy is practically an unnecessary, irrational operation. It is an unsafe surgical procedure accompanied by disastrous and fatal issues. Hysteropexy attempts to cure one lesion (excessive mobility) by producing another lesion (fixation); which is the most damaging? Its success has not warranted its continuance. Numerous post-mortems and dangerous surgical interventions have been the final story of hysteropexy.

I recall a case in which I performed an autopsy on a physician's wife with her first baby four years after hysteropexy, due directly to the operation and that only. Hysteropexy should not be performed on a child-bearingwoman. It is a passing operation, and should pass rapidly. Hysteropexy is performed on numerous neurotics, and the rest in bed for three or four weeks is what has made favorable appearances. The hysteropexy has had nothing to do with it. Vast suffering, many fatalities, and the debasement of surgery are the price of fad operations. I have found two results chiefly in hysteropexy, viz.: the uterus remains solidly fixed to the abdominal wall permanently dislocated, or more frequently the uterus becomes entirely separate from the abdominal wall, as it was before the operation. Occasionally an elongated, thin, cicatricial band (several inches) attaches the uterus to the wall. This elongated peritoneal band exposes the patient to intestinal strangulation. Women with retrodeviations practically do not need hysteropexy. They are not suffering from retrodeviations, but from some other condition, and hysteropexy is uncalled for. The naive faddist says, What are you going to do with a woman with retrodeviation? My answer is easy; I am not required to do hysteropexy—an irrational, dangerous, and unnecessary operation. In fact, I am not required to do anything with scores of retrodeviations in my practice. Those who are ill I treat for the existing pathologic condition—which is generally some inflammatory state. The uterus has no definite position, in fact; it is in its normal position when perfectly mobile. However, this is enough about pexies, though the ingenious cutting faddist is inventing new and modifying old pexies and calling it progress. The Alexander is the most rational pexy of the series, but who can find subjects to press into its service? I can find extremely few women in a year on whom it is justly applicable. It is an operation of extreme limitations. What about the surgeon who reports 1,100 Alexanders? What about the fad of finance in those four figures? He does not conduct an eleemosynary institution, but a high-priced sanitarium. At $100 a subject it would be $110,000—a retiring sum for a Chicago gynecologist.

### GASTROENTEROSTOMY.

The fad operations have now extended to that of gastroenterostomy for "ulceration of the stomach and duodenum"—just for dyspepsia.

One can scarcely believe the extent of this fad. It is rapidly spreading, and surgeons are noting with sadness how they had previously overlooked this matter. As usual, it is performed in neurotic dyspeptics. One hospital reports, for 1904, 117 operations on the stomach and duodenum, with ulcer marked in each case (and adds in some cases with complications). Gastroduodenostomy is recorded twenty-one times. Gastroenterostomy is recorded ninety times—all in one year in one hospital. Other hospitals are attempting to vie with it. Need I say that surgery has gone mad on gastroduodenal operations? The gastroenterostomy faddist asserts glibly that ulcer of the stomach, and especially of the duodenum, is a common disease, often overlooked, attended with great mortality under medical treatment, and the usual precursor of carcinoma; and that surgery offers a safe and almost sure cure of the symptoms of ulcer, besides a preventive for its complications. What are the anatomical facts in regard to this new faddism? During fifteen years at Johns Hopkins Hospital the autopsies showed that there was not 1-5 of 1 per cent. of cases presenting gastric ulcer. In Cook County Hospital I have been attending post-mortems weekly for the past twelve years, and not one duodenal ulcer was demonstrated in some 600 subjects. Also, unless the subject was affected with carcinoma of the pylorus or stomach, only two gastric ulcers were noted in 600 subjects. What sort of persons fell into the faddist's hands? I believe that hospital autopsies will present not one-half of one per cent. of gastric ulcers, and many times less of duodenal ulcer. For one gastric ulcer this would require two hundred subjects. It is easy to acquire error when gastric and duodenal ulcers are studied *in vivo*, especially when an operation is in view, and immediate report is necessary. The duodenum has two areas of color, viz.: one proximal to the exit of the biliary and pancreatic ducts, and a second quite different color distal to the exit of these ducts. Are the faddists deceived by this phenomenon? It might be advisable to follow some autopsies. That gastric ulcer is a base for carcinoma is not proved. Now comes the astonishing report that gastroenterostomy is the cure. This operation does not interfere with the ulcer. It leaves the ulcer undisturbed. How does this cure the cancer which is supposed to arise from it? Again, what are the remote effects of these scores of gastroenterostomies? Fifteen years ago I performed sixteen gastroenterostomies on dogs. I observed the remote effects of the operation, by killing the dogs at different dates subsequent to the operation. I was so indelibly impressed by the observation of those dog autopsies that I would never do gastroenterostomy on a human without ample reason (malignancy or some other insurmountable condition). In the dog the whole gastroduodenal apparatus, with the liver and pan creas, was changed, distorted, atrophied. The stomach and duodenum was a narrow contracted tube, the mucosa pale, and its folds markedly shrunken. The blood-supply was changed, and the valvular apparatus of the pancreatic and biliary ducts atrophic, and defective.

The omental adhesions were contracting and distorting the adjacent viscera. In the few human autopsies I have noted with gastroenterostomy, similar marked changes in the form, dimension, and structure were observed. The gastroduodenal and the biliary-pancreatic apparatus are very closely associated in anatomy and physiology, and to divert the food from the duodenal channel will doubtless be accompanied by evil remote effects. This article is now too long. I have no time to speak of the appendicectomy fad, the varicocele fad, the ocular myotomy fad (I saw one woman who had had the muscles of her eyes cut seventeen times, and she asserted that they were not right yet). *Quantum sufficit.—St. Louis Medical Review.*

---

### THE ERADICATION OF SYPHILIS AND GONORRHEA.

J. H. ADAMS, M.D., PAOLI, PA.

The question is arising in many medical minds whether the campaign of education in tuberculosis which is starting so nicely should not extend to venereal conditions. Certainly, if the truth were known it would seem a problem almost as serious as phthisis itself. This is the age of clamor and agitation, from Lawson down or up. Let not any sense of false modesty hold back advance in the serious work. Peterkin, an active worker in this field, is alive to this problem, and no one could hear the papers read by Kelly and Morrow at the recent meeting of the American Medical Association without echoing their conclusions. Lydston has just published a book on this subject; would that some philanthropist would put it in the hands of every young man. It is the young man that needs it; the type of girl that makes the prostitute is harder to influence, but withdraw her source of support and she ceases to be a center of pollution.

Educate the individual. Every man must make his own morals. Peterkin advises that scientific facts should be stated in a systematic, concise, yet acceptable manner, so that men and women, knowing them, will see the necessity of protecting themselves, and in so doing will limit the spread of venereal disease. These facts may be arranged in pamphlet form under the following heads: 1. Facts for all men. 2. Facts for some young men. 3. Statistics showing the dangers and monetary losses caused by venereal diseases. 4. Personal prophylaxis against venereal disease for men during sexual indulgence. 5. Personal prophylaxis against venereal disease for women during intercourse. The latter two propositions are European in idea and really defeat the others.

Until the happy day arises we must be busy with means to stamp out venereal infection whenever we find it. Baum has made a scientific study of the newer silver salts in gonorrhea with the idea of establishing their relative value; his cases being all first attacks. He followed the same plan for each, which was as follows: The patients were instructed to first urinate, then to fill up the urethra with the injection, so as to produce tension and to hold in the injected fluid from three to five minutes; the injection to be repeated five times daily, the following solution being used for these experiments: Albargin, gelatose silver, 1 to 1 per cent. solution; argyrol, 2 to 10 per cent.; argonin, casein of silver, 2 to 5 per cent.; argentamin, 1 to 3 per cent. solution; largin, silver protalbin, ¼ to 1½ per cent.; protargol, proteid of silver, ¼ to 5 per cent.; picrotol, ¼ to 1½ per cent.; the use of water in saline solution, in permanganate solution 1 to 4,000 to 1 to 500; in boric acid solution, 1 per cent. The discharge decreased quite rapidly in every case; where the silver salts were employed they suffered less in subjective symptoms and made cleaner recoveries. According to Baum those treated with argyrol were probably the most satisfactory; he believes that the organic silver salts owe their beneficial effects to bactericidal effect produced on gonococci imbedded in the upper epithelial layer, which is being exfoliated, and in preventing reinfection, rather than to their penetrating power, which he regards as small, for the prolonged pressure of any fluid on the urethral mucosa causes temporary compression of the capillaries; the removal of this pressure is followed by dilatation of these capillaries, resulting in an increased leucocytosis through the vessel walls, the point of least resistance being the lumen of the urethra, which induce more rapid exfoliation of mucous membrane cell and literally sweeps out the infection.

Valentine and Townsend read a paper at Atlantic City last June, recently published in the *Journal of the American Medical Association* that impressed me greatly as to its clearness and force; it was entitled "Gonorrhea and the General Practitioner." They are advocates of irrigation in gonorrhea, and give the following synopsis of their technique:

1. In the preponderance of cases irrigation can best be performed with the patient on a firm chair. He sits far forward, his sacrum resting on the anterior margin of the chair, the tuberosities of his ischium projecting slightly beyond.

2. The trousers are dropped to beneath the knees and the shirt and undershirt folded upward to the level of the umbilicus.

3. A clean towel is placed on the patient's thighs, covering his testicles.

4. A clean enameled or tin basin is given the patient to hold while the penis is placed over its margin.

5. The operator standing at the patient's side takes the penis with his third, fourth and fifth fingers of the left hand and supports it against the thenar eminence of the same hand, keeping his thumb and index finger free for manipulation of the gland and prepuce.

6. With the other hand he takes the irrigator's stopcock and directs the nozzle against preputial orifice.

7. By drawing back the flange of the stopcock he allows the escape of a stream of sufficient force to thoroughly cleanse the preputial orifice; subsequently the mucosa lining the preputial sac, the sulci at both sides of the frenum and finally the meatus urinarius externus. The nozzle inserted into the meatus, and the force of the flow increased to successively irrigate all parts of the anterior urethra to the compressor.

8. After irrigation the meatus is covered with a bit of absorbent cotton wet with bichlorid solution 1 to 6000, and the patient is instructed to apply fresh cotton after each urination. The sphincter vesicae being but a feeble bundle of muscular fibers, irrigation of the posterior urethra inevitably becomes an intravesical irrigation.

For posterior irrigation each of the steps before mentioned is performed, and the following added thereto: (a) After the anterior urethra has been cleansed, the nozzle is sunk into the meatus to a depth that precludes outflow of the irrigating fluid. (b) The patient is instructed to breathe deeply and to make efforts at urination. (c) The force of the flow is gradually increased until it suffices to overcome the compressor; this is appreciable to the left fingers, to whose tips resting on the urethra is communicated a purling sensation as the fluid enters the bladder. (d) The rapidity and force of the inflow grows less as the bladder is being filled, and ceases when maximum vesical distension is approached. The stopcock is then closed by thrusting forward its flange. (e) The right hand then places the stopcock within the basin, with the thumb through the ring and the fingers holding the outside of the basin, while (f) the left fingers take a glass urinal and hands it to the patient, who substitutes it for the basin as the physician removes the latter. (g) The patient empties the bladder of the solution into the glass urinal. These authorities believe the family doctor must attend these cases; if he refuses he is neglecting his duty.

Syphilis is a serious condition, and the best plan of treatment should be followed, no matter what its trouble or unpleasantness. Physicians realize this fully, and hence are apt to overdose themselves should they themselves acquire the disease.

Recently Sinclair, in the *New York Medical Journal*, calls attention to his method of treating this condition. It consists in the intramuscular injections of the salicylate of mercury; he has had very few relapses, and these he believes are due to the smallness of the dose. As to the advisability of their use as regards the patient, he writes: "I strongly advise the injections, and tell them, if they feel that the injections are too severe, I will put them on one of the other treatments. The result is that I succeed almost without exception in keeping up the injections, because the patients are amazed and pleased at the visible improvements which take place after a few treatments are given, and in case they have to go away, when they can not take the injections, and are put on inunctions or pills, they always prefer to go back to the injections; and hence I feel justified in saying that the inunction, fumigation, and internal administration of mercury are methods to be held in reserve for the patients who can not or will not be injected." Sinclair's technique is as follows ; An alcohol lamp, a sixty minim subcutaneous syringe; a needle of large caliber, which should have a high polish, a sharp point, and be about No. 19 gauge, and an inch and a half long; cotton; ether or alcohol, and collodion are necessary. The site most favorable for injection is the gluteal region, but this may be changed to the calf of the leg or the muscles of the back and chest. The part selected for the injection is bare and cleaned with a piece of cotton, moistened with alcohol or ether, and the patient holds the cotton on the spot until we are ready to inject, the object of this being twofold : First, it keeps the clothes from contaminating the spot cleaned ; and, secondly, it acts as a guide to the site of injection. The needle of the subcutaneous syringe should be thoroughly sterilized, preferably by boiling or holding it in the flame of a lamp until it is of a dull red heat. We now heat moderately the vial containing the mercury, shaking it with some vigor. The piston is withdrawn and the contents of the phial are poured into the syringe, the piston is returned and the air thus displaced. The syringe is held vertical for about a minute to let the air-bubbles rise to the base of the piston. Then, by a straight quick thrust, the needle is buried in the muscular tissue, care being taken, especially in thin subjects, not to invade the periosteum, the contents of the syringe are injected slowly and the needle is quickly withdrawn. A dry piece of cotton is placed over the injected site, and a slight gentle massage kept up for about a minute, when the puncture is sealed with cotton or collodion.

Keyes and Chetwood introduced salicylate of mercury ten years ago; it is a fine white powder insoluble in water, the dose injections being one and a half grains. It should be suspended in a heavy liquid. It comes in drachm vials each containing a grain or three-quarters with thirty minims of the liquid benzoinal.

Fournier, who is as clear a writer as he is a diagnostician, concisely analyzes the choice of treatment as follows:

A. *Ingestion by the Mouth.*—Advantages.—Simplicity and ease of administration. Disadvantages.—(1) In some cases it causes gastric trouble and diarrhea; (2) it is only tolerated in medium doses, large doses cause disturbance of digestion; (3) it is a remedy of only moderate activity and rapidity.

B. *Inunction.*—Advantages.—(1) Being an external remedy, it avoids digestive troubles; (2) for the same reason it leaves the digestive tract free for the internal administration of other drugs when required; (3) it is a remedy of great activity and rapidity. Disadvantages.—(1) It is a dirty method, and one tedious to the patient; (2) owing to the stains on linen, it can not be kept secret; (3) it is irregular—sometimes being well done, sometimes badly; (4) it causes stomatitis more than any other method.

C. *Hypodermic Injections,* daily.—Advantages.—(1) An active remedy, and one the activity of which can be regulated; (2) it avoids the digestive tract. Disadvantages.—(1) Pain, inflammation, abscess and induration. The chief of these is pain; the others are avoided by antisepsis; (2) inconvenience of daily treatment. Injections at longer intervals. Advantages.—Sometimes there is a surprising curative effect difficult to obtain otherwise, except by dangerously large doses of mercury. Disadvantages.—Pain and inconvenience. The pain is worst in calomel injections, so much so that many have abandoned this form of medication.

It can be seen from this classification that this great French syphilographer favors the use of injections, the injections being trifling considering the results. It is a serious question whether the physician is not neglecting his duty if he does not use injections wherever he can.—*Medical Fortnightly.*

---

The Wisconsin State Legislature has passed with but one dissenting vote a bill prohibiting the sale or manufacture of cigarette papers. It is not anticipated that the measure will meet with any opposition in the Senate.

---

The annual bill to abolish coroners has been presented in Albany. Such a bill has come to be an institution in the New York Legislature, so many years has it been introduced, and so many times has it "just barely missed" being passed.

### THE PSYCHOLOGICAL ASPECT OF THE DRUG HABIT.

BY FRANK F. HUTCHINS, M.D., INDIANAPOLIS, IND.

The psychological phenomena due to the abuse of drugs is to be considered from two standpoints, the temporary direct action of the poison, simple intoxication; and the more or less permanent effects resulting therefrom, mental disease. In making a distinction between the two a brief, concise consideration of the subject may best be obtained by reference to the physical basis of mind. Simple intoxication, while undoubtedly due to some brain-cell disturbance, is caused by external agencies, not at once involving the integrity of the cell. It represents the physiological action of the drug carried to excess, and may be said to rest upon a physiological process, giving the drug's peculiar hue to the intoxication. Upon the elimination of the poison the temporarily disturbed functions are shortly restored to their normal conditions. The attending stimulation and depression do not truly represent the real brain condition. They are more a characteristic manifestation of the drug. While, on the other hand, brain disease lends color to the whole mental atmosphere during the ingestion of the intoxicant and after its elimination. Intoxication is without the brain, an unusual condition; drug insanity within, the usual state for the time being. As intoxication is due practically to excessive physiological action, more than to a true pathological change in the brain itself, it does not represent the real brain status and can only properly be considered in relation to the insanity as a cause. These causes may be physical or psychical. Physical, in the action of the poison directly or indirectly upon the brain-cell; psychic, in the warping, abnormal exercise and improper use of the mental faculties during intoxication, the erratic moods and illogical association of ideas, engendered thereby, resulting in pernicious habits.

The effects of drugs are manifested in an endless variety of ways, both in different individuals and in the same individual at different times. These effects are so common and well known that comment is unnecessary, nor need they, since normal brains vary so greatly, excite wonder. It is here the role of heredity plays its far-reaching part. Every brain has born into it a certain potentiality—a power to do, an inherent force for development and cultivation—which represents its limitations. This quality of all matter is the basis of all states, the actual status of such states at a given time depending upon their potentiality, degree of development and condition of exhaustion. As potentiality is derived from the parents and development is the result of education and environment, the effects of drugs on the mind are as various and numerous as the victims of the habit. The diverse effects in the same individual at different times are accounted for by the variable resistance offered by the degree of cell exhaustion. Potentiality, degree of development and state of exhaustion in determining the brain-cell stability stand in like relation to the cause of the habit. The unfortunate becomes a victim because he has an inherent, instinctive taste or preference for the drug, undoubtedly hereditary; because of his environments, associations, or unhealthy conditions of life, or because of strain, the effort to alleviate physical or mental pain, the apparently necessary relief from overwork and excessive strife in overcoming the law of "the survival of the fittest," in the ever-increasing and intense struggle for existence. Briefly, brain-cell exhaustion. Singly or collectively do these three agencies determine the whole course of drug insanity, the beginning and the end.

The true psychoses of the drug habit are based upon rearrangement, disturbance, or want of integrity, by reason of some loss of power vitality, or reeuperative ability within the cell itself. Where this process has not extended beyond the possibility of repair, the acute insanities, similar to other forms, are most generally manifested; but where the integrity of the cell has been destroyed the grosser degenerative psychoses are more in evidence.

While there are exceptions, those faculties of mind appearing last in the scale of human evolution (not having the stability gained by their predecessors during the lapse of time) generally yield first to retrograde processes. Those finer and more delicate attributes of mind, the ethical feelings and refining influences which color and pervade all man's ideas, primarily determining his character, show the distinctive action of drugs before the older, more stable and sterner faculties of sensation and reason. It is the finer intellectual attainments, whose highly sensitive nature seems unable to resist the repeated perversions of their functions, that are particularly disturbed by intoxication, that give the cardinal principle and distinctive character to all the drug psychoses. This point is clearly illustrated by the peculiar aspect of the increasing drug habit in women. Owing to their training, and enforced application to the higher moral laws custom has established during the ages, women have approached a finer and more ethical mental plane than men. In the milder forms of the drug habit women present a moral perversion and blunting of the better nature entirely out of proportion to men, while this lack of proportion is not found in the more pronounced forms of insanity.

Following this moral degeneracy, during which the patient loses his ideas of honor and self-respect, the moods are probably the most generally affected. The individual becomes excited or depressed, erratic, morose, and easily irritated or angered. From this point an explosion may occur in any direction, or a slow and steady degeneracy may ensue. Illusions and hallucinations may arise, sometimes of a vivid character, with marked disturbances of sensation, as in delirium tremens. There may be delusions, with exultation and acceleration of the flow of ideas, constituting a true mania; or the depression and retarded thought of melancholia. Sometimes there is a pseudoparanoia, with its elaborate system of delusions, usually based on illusions and hallucinations and ideas of persecution and some grandeur. There may be amnesia, with its blurred memory-pictures and their associations, a general enfeeblement of the mental faculties; forms of paralysis, pseudoparesis, and epilepsy; in fact, all the psychoses may be simulated in drug poisoning. The process of degeneration, uninterfered with, continues until the patient sinks to the most degraded of all animals, the human brute. This general summary of the more typical forms of drug insanity is modified in various cases by the quantity and character of the drug, as well as by the individual cell characteristics.

Alcohol probably produces the most varied symptom complex, because its use is more common and extensive, its physiological action better understood, and its effect more far-reaching. Were its influences confined to the individual, or his immediate time, the necessity for its control would not be so manifest. But not only is the alcoholic's desire to procreate unduly stimulated, but the potentiality transmitted to his children is much degenerated by his excesses.

No man can offer his individual accomplishments, no matter how great or beneficial to mankind, as the full measure of his duty in life, or reason for his having existed. In the generation of children man sets in motion a germ whose possibilities in the future completely overshadow his mere selfish attainments. The parent's greatest crime is seen in the alcoholic's progeny, with their unstable, neurotic temperaments and erratic dispositions, generating in turn potentialities of crime, insanity and imbecility, until Nature uses her protective weapon, sterility, to stamp out the degenerated stock. Alcoholism knows no confining limits, masking all forms of insanity. Its only distinguishing and peculiar features are the rapid recovery from the acute manifestation under treatment and the subtle degenerative process at its base.

The use of opium in its various forms, while said to be increasing rapidly and producing the most direful results upon the mental disposition, is not to be compared to alcohol in the absolute physical changes and hereditary influences induced. Opium tends to produce a paralysis of mind, a lethargy of its faculties with abolition of will, and an inability to execute the intellectual dictations. The patient recognizes his infirmity, appreciates the better life with its higher influences, and condemns himself bitterly for his weakness in not controlling it. The slave has become master. The craving for the drug makes him stoop to all forms of dishonor. However, his former character is recognized in the great care exercised in covering and concealing the habit, and, in event of discovery, the logical and plausible theories put forward to justify the use and enable the victim to retain his social standing. The effects of opium are more noticeable in the perversions of character and ethics than in the more stormy forms of insanity. This moral disease tends in some cases to become of the very lowest forms. Nevertheless, there are other cases of long standing in which the mental faculties do not seem to suffer. The degenerative effects of opium are slow and peculiarly characteristic. Opium seems more a food than a poison. This is evinced in its treatment, for patients with well-developed habits apparently make good recoveries. Except in the worst forms the effects upon the progeny are not so marked. But as the opium habitue seeks his solitary enjoyment, and the sexual instincts are diminished, positive indications of the transmitted potentiality are not so evident.

The use of cocaine is dying out, probably as much from the annihilation of its votaries as from a lessening tendency toward its use. The cocaine habit is an insidious one, disastrous in its results. It produces a mental craving whose satiation requires rapidly increasing doses. The large doses, combined with the potency of the drug, soon produce marked physical changes and a rapid transition from simple intoxication to true mental disease. Cocaine insanity is characterized by the violence of its manifestations. These tend toward the most aggravated varieties of manias of persecutory and paranoia-like forms.

There are habits of chloral, chloroform, hyoscyamus, hashish, coffee, tobacco, and a host of other forms of poisonings or intoxications. Each has its votaries and shows its more or less characteristic or peculiar action. The description of the manifestation of these intoxications might go on ad infinitum, but, after all, it would be insignificant in comparison with the real root or basic principle underlying the whole subject of drug insanity. It matters not whether a man suicides by hanging or poisoning, the attained end is just the same, the method is merely incidental, the real problem being the reason for its employment. The tendency of the times is to consider the methods, to condemn the innocent drugs, whose evil effects are merely a force exerted in a wrong direction. This force properly directed might have an equal capacity for good.

If ever the drug habit is to be controlled a real basis for treatment must be sought in the hereditary, environment and strain of the individual. As long as the world offers such rewards for supremacy, and civilization continues to tolerate the parasitical operations of the weak, men will continue to strive and strain beyond their capacity, seeking at times of crises to condense their energies, or blunt the sting of the crisis by artificial stimulation. The form of stimulant can easily be duplicated, and the elimination of them all, presuming it possible, would remove a potent force for good. Might not a remedy be found in a change from the present artificial to a more natural life, and the denial to the unfit of transmitting defective potentiality? These are two important factors in establishing a more equal ratio between man's ambitions and his physical capacity.—*Medical and Surgical Monitor*.

---

We learn from an exchange that hereafter license to practice medicine in North Dakota will be awarded on presentation of a diploma granted by a college giving four courses of lectures of at least eight months each and an examination in prescribed subjects. The board is given power to revoke or refuse a license for dishonorable or immoral conduct, chronic or persistent inebriety or mental aberration, excessive use of narcotics, or for the practice of criminal abortion. In complaints for violating the provisions of this section of the law the accused must be furnished with a copy of the complaint, and be given a hearing before the board in person or by an attorney. The law provides for reciprocity as follows: The board in its discretion may grant license for the same fee ($20) without examination to applicants examined and licensed by other State examining boards maintaining standards not lower than those provided for by the law.

---

A Tuberculosis Directory is published jointly by the Charity Organization Society and the National Association for the Study and Prevention of Tuberculosis. The names of American institutions, many statistics, and much information for both the poor and the well-to-do, for both physicians and patients, are compressed within 250 pages.

